# Intracranial antitumor responses of nivolumab and ipilimumab: a pharmacodynamic and pharmacokinetic perspective, a scoping systematic review

**DOI:** 10.1186/s12885-019-5741-y

**Published:** 2019-05-30

**Authors:** Mark T. J. van Bussel, Jos H. Beijnen, Dieta Brandsma

**Affiliations:** 1 Division of Pharmacology, Netherlands Cancer Institute, Antoni van Leeuwenhoek, Plesmanlaan 121, 1066 CX Amsterdam, The Netherlands; 2 Department of Medical Oncology & Clinical Pharmacology, Netherlands Cancer Institute, Antoni van Leeuwenhoek, Plesmanlaan 121, 1066 CX Amsterdam, The Netherlands; 3 Department of Pharmacy & Pharmacology, Netherlands Cancer Institute – Antoni van Leeuwenhoek, Plesmanlaan 121, 1066 CX Amsterdam, The Netherlands; 4 Department of Pharmaceutical Sciences, Division of Pharmacoepidemiology & Clinical Pharmacology, Faculty of Science, Utrecht University, Universiteitsweg 99, 3584 CG Utrecht, The Netherlands; 5 Department of Neuro-oncology, Netherlands Cancer Institute - Antoni van Leeuwenhoek, Plesmanlaan 121, 1066 CX Amsterdam, The Netherlands

**Keywords:** Melanoma brain metastases, Nivolumab, Ipilimumab, Neonatal fc receptor, Pharmacodynamics, Pharmacokinetics

## Abstract

**Background:**

Recently, two phase II trials showed intracranial activity of the immune checkpoint inhibitors nivolumab and ipilimumab in patients with melanoma brain metastases. However, it is generally assumed that large molecules like monoclonal antibodies nivolumab and ipilimumab cannot penetrate and pass an intact blood brain barrier (BBB). In this systematic review we provide a pharmacodynamic and pharmacokinetic consideration of the clinical activity of the immune checkpoint inhibitors nivolumab and ipilimumab in melanoma brain metastases.

**Methods:**

Pubmed was systematically searched for prospective phase II and III studies on nivolumab and ipilimumab in melanoma brain metastases and cerebrospinal fluid (CSF) levels of nivolumab and ipilimumab. Results were discussed and a perspective on the pharmacodynamics and pharmacokinetics for the intracranial activity of these agents was given.

**Results:**

Two phase II studies with the combination nivolumab and ipilimumab and one phase II study with ipilimumab monotherapy in melanoma brain metastases were included in this review. One article reported drug levels of nivolumab in CSF. Intracranial responses were achieved in 16 of 35 patients (46%; 95% confidence interval (CI) 29–63) in a phase II study cohort treated with nivolumab and ipilimumab. In a second phase II study in 94 patients, the rate of intracranial clinical benefit was 57% (95% CI 47–68). The CSF/serum ratio of nivolumab was 0.88–1.9% in a cohort of metastatic melanoma patients treated with nivolumab 1–3 mg/kg. Nivolumab concentrations ranged from 35 to 150 ng/ml in CSF of these patients, which is in the range of the half maximal effective concentration (EC50) of 0.64 nM.

**Conclusions:**

Ipilimumab and nivolumab are active in melanoma brain metastases. Nivolumab penetrates into the CSF. Based on the described findings the general consensus that monoclonal antibodies do not penetrate into the central nervous system (CNS) and cannot have a direct intracranial effect needs to be reconsidered.

## Background

Immunotherapy with immune checkpoint inhibitors has become first line therapy in patients with metastatic melanoma [1]. Nivolumab (MDX-1106) is a human immunoglobulin G4 (IgG4) monoclonal antibody which binds to the programmed death-1 (PD-1) receptor and blocks its interaction with PD-L (programmed death ligand) 1 and PD-L2 [2]. Activation of the PD-1 receptor inhibits T cell activity which is important in the inhibition and thus regulation of T cell immune responses. PD-L1 and PD-L2 are expressed by antigen presenting cells and can be expressed by tumors cells [3, 4]. Nivolumab potentiates T cell responses against tumor cells through blockade of PD-1 receptor binding to PD-L1 and PD-L2. Ipilimumab is a fully human anti-cytotoxic T lymphocyte associated antigen 4 (CTLA-4) IgG1κ monoclonal antibody [5]. CTLA-4 present on activated T cells can induce T cell inhibitory signals [6]. The combination of intravenous nivolumab and ipilimumab had a higher efficacy than intravenous nivolumab monotherapy in a randomized, double-blind, phase III study with 945 previously untreated patients with unresectable stage III or IV melanoma [7]. The general consensus with regard to antibody pharmacokinetics is that monoclonal antibodies cannot penetrate an intact BBB due to their large molecular size and thereby may lack clinical activity in the CNS [8–13]. However, the BBB of blood vessels in brain metastases is partially disrupted leading to a higher permeability [14]. Recently, two phase II studies have shown intracranial efficacy of nivolumab and ipilimumab in patients with melanoma with untreated brain metastases [15, 16]. With regard to the highly promising intracranial effects of immune checkpoint inhibitors administered intravenously in melanoma patients with brain metastases, we would like to give a perspective of the pharmacokinetics and pharmacodynamics on the intracranial antitumor activity of nivolumab and ipilimumab. In this paper, we argue against the consensus that monoclonal antibodies such as immune checkpoint proteins inhibitors cannot penetrate an intact BBB and thereby cannot be efficacious against CNS tumors via this direct intracranial mechanism. We show a concise mechanistic insight on the pharmacodynamics of the intracranial activity of nivolumab and ipilimumab.

### The immune system in brain metastases

One of the characteristics of the CNS is the lack of a classical lymphatic drainage system. However, based on recent research, it is now accepted that the CNS undergoes constant immune surveillance within the meningeal compartment [17–19]. Soluble antigens derived from tumors within the CNS can reach the deep cervical lymph nodes via CSF drainage. Antigen presenting cells take up neo-antigens from the intracranial tumor and present them in the cervical lymphnodes to lymphocytes. To mediate a pharmacodynamic therapeutic effect in the brain, the systemically activated effector immune cells or the checkpoint inhibiting antibody has to reach the intracranial tumor site. Lymphocytes activated in the cervical lymph node can enter the brain and CSF via the blood. Tumor cells are able to evade these activated lymphocytes by expressing PD-L1 to inhibit the activated T cell. Tumor infiltrating lymphocytes (TILs) are present in melanoma brain metastases [3, 20, 21]. In a retrospective cohort of 43 melanoma brain metastases CD3+ TILs were present in 77% of the samples and CD8+ TILs were present in 91% of the samples [3]. Fifty-one percent of melanoma brain metastases expressed PD-L1 [3]. The ligand and the effector immune cells are thus present in the tumor brain environment.

## Methods

First Pubmed was searched using the following terms: nivolumab OR ipilimumab OR nivolumab AND ipilimumab AND melanoma brain metastases NOT radiotherapy up to 24 December 2018. Prospective phase II-III studies in melanoma brain metastases were included. Modified Response Evaluation Criteria in Solid Tumors (RECIST) criteria for brain lesions or modified WHO response criteria were extracted from the clinical studies to assess efficacy. A second search was performed for (ipilimumab AND cerebrospinal fluid) OR (nivolumab AND cerebrospinal fluid) to identify additional papers in which CSF levels of nivolumab or ipilimumab are reported.

## Results

**Fig. 1:**
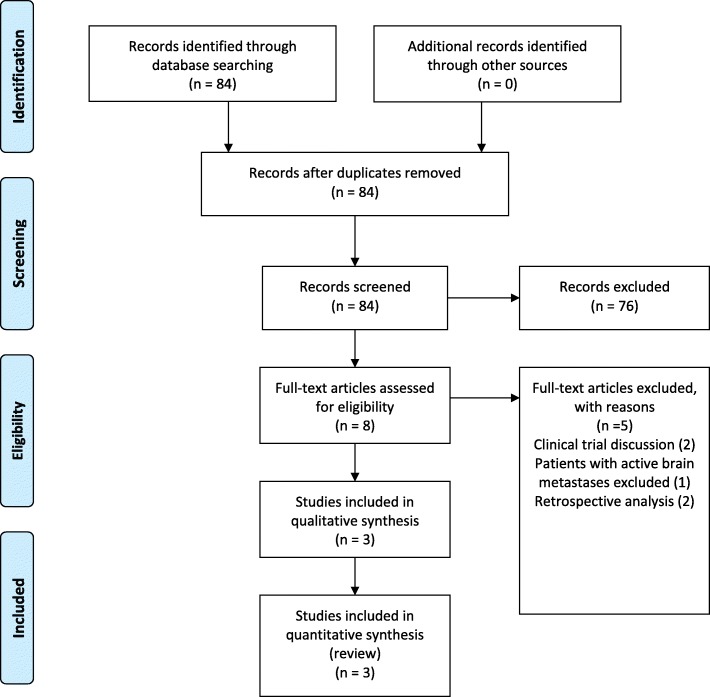
PRISMA diagram of clinical study selection

**Fig. 2:**
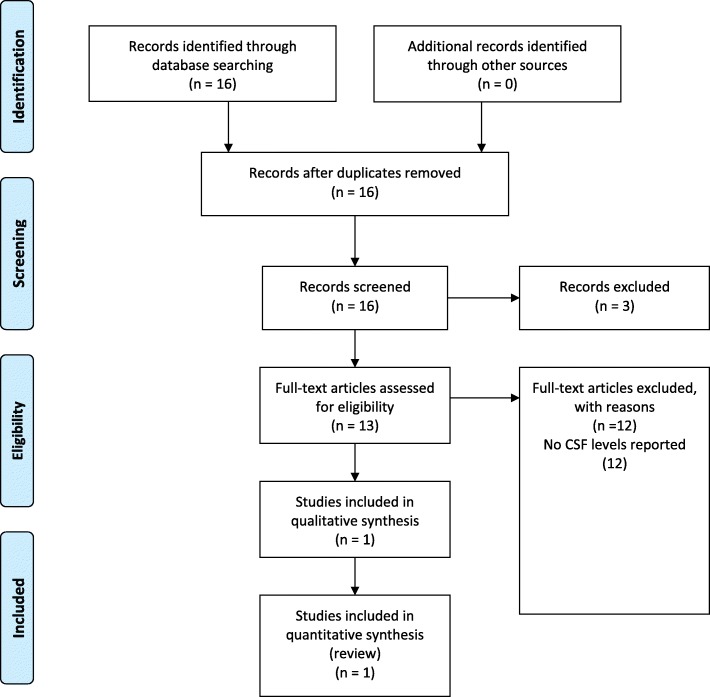
PRISMA diagram of study selection with reported CSF levels for nivolumab or ipilimumab

The Pubmed search resulted in 84 hits. Two prospective phase II studies with the combination nivolumab and ipilimumab and one prospective phase II study with ipilimumab monotherapy in melanoma brain metastases were included for this review as shown in Fig. 1. No nivolumab or ipilimumab levels in CSF were reported in the phase II studies. Therefore, a second Pubmed search was performed specifically for nivolumab and CSF or ipilimumab and CSF. The search resulted in 16 hits as shown in Fig. 2. One article was found in which drug levels of nivolumab in CSF were reported. No articles reported ipilimumab levels in CSF.

### Clinical studies

In the first clinical study 63 immunotherapy-naïve patients with asymptomatic brain metastases were randomized to intravenous nivolumab 1 mg/kg combined with intravenous ipilimumab 3 mg/kg every 3 weeks for four doses and were subsequently treated with nivolumab 3 mg/kg every 2 weeks in cohort A or nivolumab 3 mg/kg every 2 weeks patients in cohort B [16]. In the non-randomized cohort C, patients with progressive brain metastases after local therapy, patients with symptomatic brain metastases or with leptomeningeal disease were treated with nivolumab 3 mg/kg every 2 weeks. The primary endpoint was intracranial response defined as the percentage of patients with a confirmed intracranial complete or partial response at week 12. At the data cutoff with a median follow up of 17 months (IQR 8–25) intracranial responses were achieved by 16 of 35 patients (46%; 95% CI 29–63) in cohort A, five of 25 (20%; 95% CI 7–41) in cohort B and one of 16 (6%; 95% CI 0–30) in cohort C. The median intracranial progression free survival has not been reached in cohort A. The intracranial progression-free survival at 6 months was 53%.

In the second clinical study, 94 patients with metastatic melanoma and at least one measurable, non-irradiated asymptomatic brain metastasis received nivolumab (1 mg/kg) plus ipilimumab (3 mg/kg) every 3 weeks for up to four doses, followed by nivolumab (3 mg/kg) every 2 weeks until progression or unacceptable toxicity [15]. The primary endpoint was the rate of intracranial clinical benefit, defined as the percentage of patients who had stable disease for at least 6 months or a partial response or a complete response. At a median follow-up of 14 months the rate of intracranial clinical benefit was 57% (95% CI 47–68), the complete intracranial response rate was 26% and the partial intracranial response rate was 30%. This resulted in an intracranial objective response of 55% (95% CI 45–66). The median duration of intracranial response has not been reached.

Ipilimumab monotherapy has been studied in a phase II study in 72 patients with melanoma and brain metastases [22]. Patients received four doses of 10 mg/kg intravenous ipilimumab once every 3 weeks. Patients who were clinically stable at week 24 were eligible to receive 10 mg/kg ipilimumab every 12 weeks. Patients in cohort A were neurologically asymptomatic and were not receiving corticosteroids at inclusion. Patients in cohort B were symptomatic and received a stable dose of corticosteroids. The primary endpoint was the proportion of patients with disease control, defined as complete response, partial response or stable disease after 12 weeks, assessed with modified WHO criteria. CNS disease control assessed in 51 patients in cohort A was 24% (95% CI 13–38) and 10% (95% CI 1–30) in cohort B, which consisted of 21 patients.

The above mentioned clinical trials clearly demonstrated intracranial responses of patients with melanoma brain metastases treated intravenously with immune checkpoint inhibitors. Four other clinical trials with nivolumab and ipilimumab in patients with melanoma brain metastases are ongoing [23]. In a phase II trial nivolumab and ipilimumab is combined with radiotherapy (NCT03340129). In a phase III trial nivolumab and ipilimumab are combined with fotemustine (NCT02460068). Recently, a phase I/Ib trial (NCT03025256) of concurrent intravenous and intrathecal nivolumab for patients with leptomeningeal metastases has started.

### Nivolumab pharmacodynamics

Nivolumab is a human IgG4 monoclonal antibody. IgG4 antibodies can undergo Fab (Fragment antigen binding)-arm exchange [24, 25]. Fab-exchange can be prevented by introducing a mutation in the hinge region of the antibody, as has been done for nivolumab [25, 26]. The constant region fragment (Fc) of the antibody determines the effector functions and kinetics [27]. Antibodies with neonatal Fc receptor (FcRn) binding can enter cells via endocytosis and are prevented from degradation by the FcRn, resulting in a prolonged elimination half-life [27–29]. Nivolumab is an IgG4 antibody with FcRn binding [30]. IgG4 antibodies like nivolumab have a low potential to induce antibody dependent cell mediated cytotoxicity (ADCC) or complement dependent cytotoxicity (CDC) [27, 30]. This prevents toxic effects of nivolumab on the lymphocytes themselves and thereby preserves T cell function. Nivolumab binds to native PD-1 molecules expressed on activated T cells with an EC50 of 0.64 nM [30]. No dose response relation has been observed in melanoma patients treated with intravenous nivolumab dosed from 0.1 to 10 mg/kg. The receptor occupancy of nivolumab has been investigated at a dose range from 0.1–10 mg/kg. The median PD-1 receptor occupancy by nivolumab was 64–70% across all dose levels. These results demonstrate that the majority of PD-1 receptors are bound by nivolumab at the lowest dose level tested (0.1 mg/kg). No effect between dose and receptor occupancy was observed within the studied dose range. A sustained receptor occupancy above 70% of nivolumab on PD-1 on circulating T cells has been observed for more than 2 months after nivolumab infusion despite a serum half-life of nivolumab of 12 to 20 days regardless of dose [26].

### Nivolumab pharmacokinetics

Nivolumab is dosed intravenously and has linear pharmacokinetics within the studied dose range of 0.1–10 mg/kg [2]. Based on population pharmacokinetic analysis at steady state at dose level 3 mg/kg every 2 weeks, the clearance, terminal half-life and average exposure were 7.9 ml/h, 25.0 days and 86.6 μg/ml, respectively. The registered dose interval was initial biweekly. However, in melanoma and in renal cell carcinoma the dose interval has been doubled to 4 weeks based on modelling of dose/exposure efficacy and safety relationships [2]. The molecular weight of nivolumab is 146 kDa [31]. As stated earlier, the EC50 of nivolumab binding to native PD-1 molecules expressed on activated T cells is 0.64 nM [30]. In a cohort of metastatic melanoma patients with a clinical suspicion on leptomeningeal metastases, treated with nivolumab 1–3 mg/kg every 2–3 weeks, nivolumab CSF levels have been quantified with a validated enzyme-linked immunosorbent assay [32]. The nivolumab concentrations ranged from 35 to 150 ng/ml with a CSF/serum ratio of 0.88–1.9% [32]. The CSF levels of nivolumab are in the range of the EC50 with a molar range of 0.24–1.0 nM.

### Ipilimumab pharmacodynamics

CLTLA-4 induces T cell inhibitory signals [6]. CTLA-4 is transiently expressed by a subset of activated T cells and binds to the B7 (CD80/CD86) receptor on antigen presenting cells [33, 34]. Ipilimumab is an IgG1κ anti-CTLA-4 monoclonal antibody [5]. Ipilimumab has an EC50 of 0.2 μg/ml for the in-vitro binding of human CTLA-4 to B7.1 (CD80) and B7.2 (CD86) with maximal blockage between 6 to 20 μg/ml and 1 to 3 μg/ml respectively [33]. The target trough concentration for ipilimumab is 20 μg/ml based on in vitro studies. Intravenous ipilimumab induces a dose dependent increase in absolute lymphocyte counts (ALC) [5, 35]. This includes an increase of central memory CD4- and CD8 T cells and effector memory T cells [5]. Given the rise in ALC it is unlikely that ADCC does occur at biological relevant levels during ipilimumab treatment [33]. Partial and complete ongoing systemic responses of ipilimumab have been observed till months after the last ipilimumab administration [36]. The persisted pharmacodynamic effects have been maintained by the immune system in the absence of therapeutic ipilimumab concentrations for more than 710 days [36].

### Ipilimumab pharmacokinetics

Ipilimumab has dose proportional pharmacokinetics over the dose range of 0.3 mg/kg to 10 mg/kg [33]. The terminal half-life is 15.4 days [5]. Ipilimumab accumulates due to the dose interval of once every 3 weeks and an estimated elimination half-life of 2 weeks. The molecular weight of ipilimumab is 148 kDa [33]. Ipilimumab has a systemic clearance of 16.8 ml/h (percent coefficient of variation) (38.1%) and a volume of distribution of 7.47 l (10.1%) at steady-state. The average steady state trough serum concentration (±SD) of ipilimumab was 21.8 μg/ml (± 11.2) at the 3 mg/kg induction regimen.

The FcRn binding properties of ipilimumab have not been assessed [37]. However, IgG1 binds to the FcRn and therefore it is assumed that ipilimumab also binds to the FcRn [27]. CTLA-4 immune checkpoint inhibitors block T cell inhibitory signals induced by the CTLA-4 pathway and increases the number of reactive T effector cells, which induce a direct T cell immune attack against tumor cells [5, 35]. CTLA-4 blockade can also inhibit the function of regulatory T cells which may provide an antitumor immune response. As ipilimumab is an IgG1 monoclonal antibody, it is expected that ipilimumab can also reach the CSF via FcRn mediated transcytosis. However, to the best of our knowledge, no studies have been published on ipilimumab concentrations in CSF.

## Discussion

**Fig. 3:**
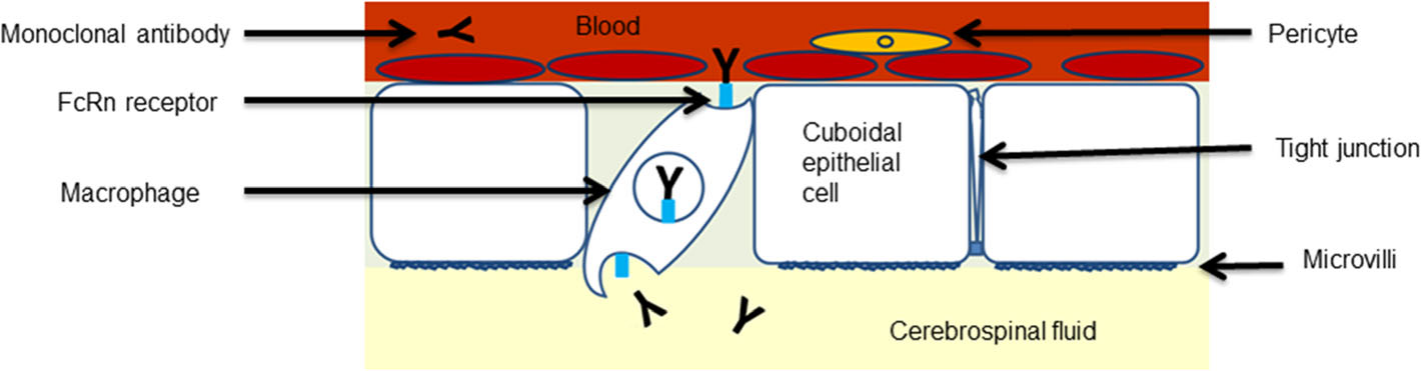
Proposed mechanism of nivolumab transport to the CSF. Neonatal Fc receptor mediated antibody transport from the blood vessels in the choroid plexus to the CSF. The choroid plexus consists of a monolayer of cuboidal epithelial cells in which macrophages reside [41]. The epithelial cells have microvilli and are interconnected via tight junctions forming the blood-CSF barrier. The proposed mechanism of FcRn mediated endocytosis of IgG4 monoclonal antibodies with FcRn binding like nivolumab is via macrophages residing in the choroid plexus [29, 41]. Monoclonal antibodies with FcRn binding like nivolumab are prevented from degradation by the FcRn [29]. The FcRn mediates antibody transport to the CSF. FcRn neonatal Fc receptor, CSF cerebrospinal fluid, IgG4 immunoglobulin G4

Clinical studies in the recent years have shown a high intracranial effect of the combination of ipilimumab and nivolumab (55% intracranial response) on melanoma brain metastases, which is considered to be mediated by an intracranial increase of activated lymphocytes by blocking the two immune checkpoint proteins on T cells [15, 16]. The general consensus on the intracranial effect of immune checkpoint inhibitors is that the expected pharmacodynamics effect is caused by activated peripheral T cells which then cross the BBB. Monoclonal antibodies are not believed to penetrate an intact BBB due to their large molecular size of ∼150 kDa [8–12, 38]. A sustained receptor occupancy above 70% of nivolumab on PD-1 on circulating T cells has been observed for more than 2 months after infusion, despite a serum half-life of nivolumab of 12 to 20 days regardless of dose [26]. Nivolumab can bind the peripheral circulating T cells irreversibly which then cross the BBB. The antitumor effect of nivolumab is mediated by activated T cells given the low potential of nivolumab to induce ADCC or CDC activity [27, 30]. However, both nivolumab and ipilimumab are IgG monoclonal antibodies with FcRn binding, which can cross an intact BBB. FcRn binding of antibodies is known to mediate transport of IgG antibodies over the placenta from mother to child and is involved in other transcellular transport processes [39, 40]. IgG antibodies with FcRn binding like nivolumab can enter cells, like macrophages in the choroid plexus and reach the CSF via endocytosis via FcRn mediated transcytosis [41, 42]. 80% of the total CSF production of 500–600 ml per day is produced by the choroid plexus via filtration of the blood [41]. Microglial cells, macrophages and dendritic cells reside in the choroid plexus and can mediate the nivolumab transport of the blood to the CSF. The proposed mechanism of nivolumab transport via the choroid plexus to the CSF is depicted in Fig. 3.

The FcRn mediated transcellular transport is a saturable system [38]. High IgG concentrations will increase the antibody fraction that is being catabolized, leading to a decrease in the elimination half-life. IgG has an elimination half-life of 25 days and a plasma clearance of 10 ml/h [38]. IgG4 is the least common antibody subclass in of the IgG subclasses 1–4 with a serum concentration of 0.5 mg/ml [43]. Nivolumab has a clearance of 7.9 ml/h [2]. This indicates that the FcRn receptor mediated transcytosis has not yet been saturated at therapeutic concentrations and that the maximum physiological capacity of this transport system has not been reached [27, 28]. The combination of nivolumab and ipilimumab has a higher systemic efficacy than monotherapy in a randomized, double-blind, phase III study with 945 previously untreated patients with unresectable stage III or IV melanoma [7]. The systemic response rate for nivolumab monotherapy was 43.7% (95% CI 38.1–49.3), for ipilimumab monotherapy 19.0% (95% CI 14.9–23.8) and for the combination 57.6% (95% CI 52.0–63.2). In the CNS, a similar increase of antitumor activity has been observed for the combination of nivolumab and ipilimumab, with intracranial responses of 46% (95% CI 29–63) for the combination of nivolumab and ipilimumab and 20% (95% CI 7–41) for nivolumab monotherapy [16]. Currently, the pharmacodynamic effect of ipilimumab is considered to be on the peripheral T cells which then cross the BBB [10]. The sustained pharmacological effect of ipilimumab can be attributed to an increase of central memory CD4 and CD8 T cells and effector memory T cells [5]. Whether an additional, direct effect of ipilimumab in the brain occurs is unknown, as no data are available on ipilimumab FcRn binding and ipilimumab concentrations in the CSF.

## Conclusions

Based on the described findings the general consensus that monoclonal antibodies do not penetrate into the CNS and that this mechanism does not contribute to intracranial activity of these agents needs to be reconsidered. The intracranial effects of immune checkpoint inhibitors can be due to a dual mechanism: they can bind irreversibly PD1 or bind to CTLA-4 on peripheral circulating lymphocytes which can subsequently penetrate the BBB (mechanism 1) and the antibodies themselves can cross the BBB and inhibit the TILs, being already present in the intracranial tumor (mechanism 2). For adequate brain penetration of antibodies, they need to be selected for the optimal IgG subclass with FcRn binding and favorable pharmacokinetics in the early drug development process. The highly promising clinical antitumor activity combined with the described mechanism of penetration of monoclonal antibodies into the CSF opens novel strategies to treat malignant diseases in the CNS.

## Data Availability

All data generated or analysed during this study are included in this published article.

## Abbreviations

ADCC: Antibody dependent cell mediated cytotoxicity
BBB: Blood brain barrier
CDC: Complement dependent cytotoxicity
CI: Confidence interval
CNS: Central nervous system
CSF: Cerebrospinal fluid
CTLA-4: cytotoxic T lymphocyte associated antigen 4
EC50: Half maximal effective concentration
Fab: Fragment antigen binding
Fc: Constant region fragment
FcRn: Neonatal Fc receptor
IgG4: Immunoglobulin G4
PD-1: Programmed death-1
PD-L: Programmed death ligand
RECIST: Response Evaluation Criteria in Solid Tumors
TILs: Tumor infiltrating lymphocytes
